# Non-Invasive Imaging for the Diagnosis of Genital Warts and Their Imitators

**DOI:** 10.3390/jcm13051345

**Published:** 2024-02-27

**Authors:** Elisa Cinotti, Lorenzo Barbarossa, Giulio Cortonesi, Arianna Lamberti, Francesca La Marca, Linda Tognetti, Pietro Rubegni, Jean Luc Perrot

**Affiliations:** 1 Dermatology Section, Department of Medical, Surgical, and Neurological Sciences, Santa Maria Alle Scotte Hospital, 53100 Siena, Italy; elisa.cinotti@unisi.it (E.C.); giuliocortonesi@gmail.com (G.C.); ariannalamberti@virgilio.it (A.L.); francescalamarca93@gmail.com (F.L.M.); linda.tognetti@dbm.unisi.it (L.T.); pietro.rubegni@gmail.com (P.R.); 2 Department of Dermatology, University Hospital of Saint-Etienne, 42100 Saint-Etienne, France; j.luc.perrot@chu-st-etienne.fr

**Keywords:** wart, genital, condyloma, non-invasive, imaging, molluscum contagiosum, Fordyce’s spot, lymphangioma

## Abstract

Genital warts are the most frequent sexually transmitted disease. Their clinical diagnosis is not always easy, and invasive skin biopsies for histological examination should be performed in these cases. The aim of the study was to investigate the use of non-invasive imaging techniques for the diagnosis of genital warts and their imitators. We retrospectively evaluated dermoscopy, reflectance confocal microscopy (RCM), and line-filed confocal microscopy (LC-OCT) images of nine patients with 19 warts of the mucous membranes and five patients with lesions that clinically mimic genital warts, including 12 molluscum contagiosum, 1 Fordyce’s spot and one case of multiple acquired lymphangiomas. Most genital warts (15; 79%) showed dilated vessels surrounded by a whitish halo at dermoscopy. RCM and the new device LC-OCT could identify near histologic features such as the presence of hyperkeratosis, acanthosis, papillomatosis and enlarged vessels in all genital warts. However, the identification of koilocytes, which are the hallmark for the diagnosis of warts, was still difficult using both techniques. Non-invasive imaging techniques could also offer clues for the correct diagnosis of the imitators. This study confirmed the usefulness of dermoscopy in recognizing a precise pattern in warts and showed the potential use of RCM and LC-OCT to add additional findings to the clinical and dermoscopic examination.

## 1. Introduction

Sexually transmitted infections (STDs) resulting from the human papillomavirus (HPV) are known as genital warts or condyloma acuminata. It is categorized as a worldwide epidemic and is thought to be the most common STD found in poor nations [1]. HPV infection affects mucosal epithelial cells and keratinocytes, causing anogenital and cutaneous warts, mostly of a benign and self-limiting nature. Genital warts present as flat, sessile, or pedunculated flesh-color, whitish, or more rarely pigmented lesions. The diagnosis is often clinical, without the need for non-invasive imaging and/or a histological examination. However, noninvasive imaging techniques such as dermoscopy, reflectance confocal microscopy (RCM), and colposcopy, after the application of 3–5% of acetic acid [2], can be used to confirm the clinical diagnosis and exclude other diseases. Moreover, line-field confocal optical coherence tomography (LC-OCT), a new imaging technique, could be promising in this field. We described dermoscopy, RCM, and LC-OCT features of a series of warts of the mucous membrane and of a series of lesions that were clinically mistaken for genital warts.

## 2. Materials and Methods

The aim of the study was to investigate the use of non-invasive imaging techniques, including dermoscopy, LC-OCT, and RCM, for the diagnosis of genital warts and their imitators. All available dermoscopic, LC-OCT, and RCM images of HPV-induced lesions of the mucous membranes and their imitators acquired from 1 January 2021 to 1 December 2023 were retrospectively collected from the database of the Dermatology Departments of the University Hospital of Siena (Italy) and the University Hospital of Saint-Etienne (France). Inclusion criteria were age of the patient >18 years and histopathological examination available for at least one lesion per patient. Cases with images of poor quality judged by an expert on non-invasive skin imaging (E.C.) were excluded. The study was conducted according to the criteria set by the Declaration of Helsinki. All data were deidentified before use.

### 2.1. Dermoscopy

Dermoscopic images were acquired with Viosiomed (MAVIG GmbH, Munich, Germany) and the dermoscope coupled with the LC-OCT device at 20× magnification.

### 2.2. LC-OCT

An LC-OCT device (LC-OCT; DeepLive^®^, DAMAE Médical, Paris, France) was used to capture vertically oriented images/videos and 3D images. The system offers 10 frames/second acquisition rate and has an axial resolution of 1.2 μm, a lateral resolution of 1.3 μm, a scanning depth of 500 μm, and a lateral field of view of 1.2 mm [3]. The device provides a dermoscopic image of the area that is imaged by LC-OCT and can provide images of the entire lesions at 20× magnification.

### 2.3. RCM Device

The hand-held VivaScope 3000^®^ camera (MAVIG GmbH, Munich, Germany) was used for the in vivo RCM examination. It uses an 830 nm wavelength laser and produces images with an axial resolution of 3–5 μm and an axial resolution of 1 μm, which correspond to a horizontal section of the skin measuring 920 μm × 920 μm up to 250 μm in depth.

### 2.4. Image Acquisition and Evaluation

To guarantee that the refractive indices matched, a drop of paraffin oil was inserted between the lesions and the camera lens for the LC-OCT and RCM examination. For each lesion, at least four 2D and one 3D LC-OCT images, one LC-OCT video, and at least three RCM images of various depths (epidermis, DEJ, and dermis when permitted by a not-excessive thickness of the epidermis) considered pertinent for the diagnosis were obtained. All study lesions were acquired by two dermatologists with more than 10 years of experience in skin imaging (E.C. and J.L.P.) and evaluated by one of these experts (E.C.). Data were recorded in an Excell file (Microsoft Excel 2019).

## 3. Results

Nine patients (three women and six males; mean age 47 years, standard deviation 10.5 years, range 30–61 years) with 19 warts (mean 2 warts per patient, range 1–4) and five patients (three women and two males; mean age 43 years, standard deviation 12 years, range 23–52 years) with lesions that were in clinical differential diagnosis with genital warts) were included in the study. Two women and a man had 12 molluscum contagiosum (13 lesions with mean 4 lesions per patient, standard deviation 3.6, range 1–8), one woman had a Fordyce’s spot, and one man had uncountable multiple lymphangiomas (Table 1).

### 3.1. Images of the Genital Warts

Seven warts were in the pubic area, four on the penis, three on the labia majora, two on the perianal area, one on the inferior lip, and two on the inguinal folds. The lesions from the same patients had similar clinical, dermoscopic, RCM, and LC-OCT findings. Dermoscopy was available in all cases, RCM was available for seven warts, and LC-OCT in eleven cases.

**Figure 1:**
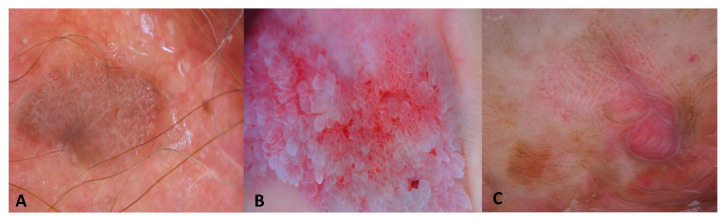
Dermoscopy patterns of condylomas. (**A**) Condyloma of the internal face of the labia majora presents a mosaic pattern with dotted vessels and pigmented homogeneous brown areas. (**B**) Condyloma of the perianal region with fingerlike projections centered by glomerular vessels. (**C**) Condyloma of the periurethral orifice showing unspecific pattern with dotted and linear vessels.

Dermoscopy revealed the presence of repetitive small structures formed by a central vessel surrounded by a whitish halo in most of the patients (15; 79%, Figure 1). Thirteen warts presented with a confluent mosaic pattern consisting of a whitish network circumscribing rounded areas centered by dilatated dotted or glomerular vessels (Figure 1). One patient (patient number 8 in Table 1) had three condylomas of the perianal area that showed multiple papillomatous projections (finger-like pattern) that contained linear vessels (Figure 1). One patient (patient number 9 in Table 1) had an unspecific dermoscopic pattern in the per-urethral area (Figure 1).

**Figure 2:**
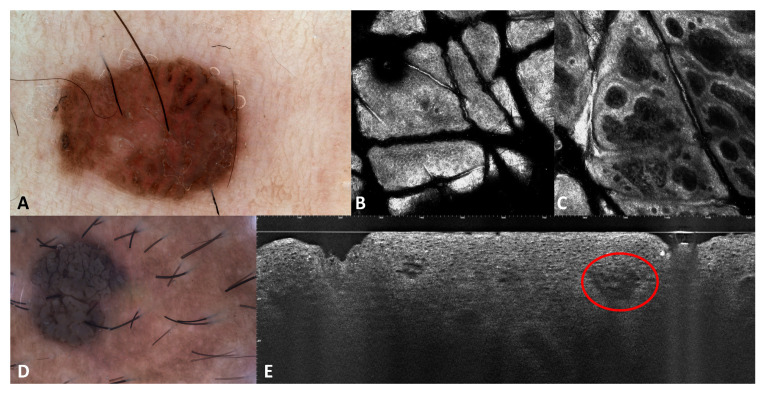
Dermoscopic (**A**–**D**), RCM (**B**,**C**), and LC-OCT (**E**) images of pigmented warts. The red circle on LC-OCT images indicates dilatated superficial vessels.

One phototype III patient (patient number 3 in Table 1) and one phototype IV (patient number 6 in Table 1) patient presented with pigmented genital warts (Figure 2). In two additional prototype III patients (patients number 1 and 2 in Table 1), condylomas had focal areas of pigmentations. The phototype IV (patient number 6 in Table 1) patient had two genital warts with a homogenous brown pattern and the phototype III patient (patient number 3 in Table 1) had a single wart in the pubic area with a homogenous brown pattern and slightly visible linear vessels.

**Figure 3:**
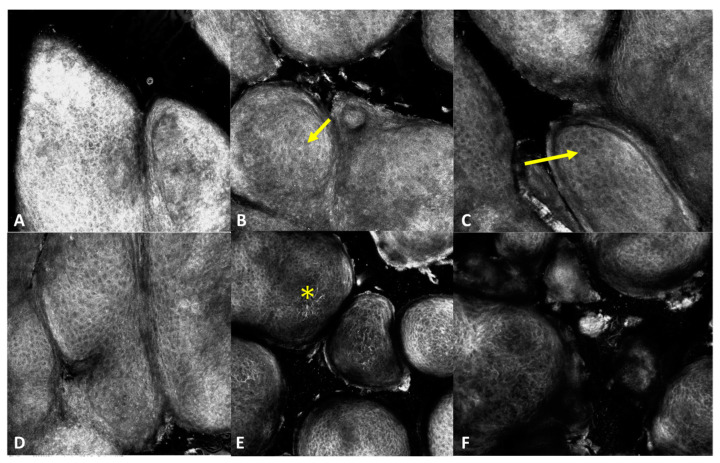
RCM images of condyloma (**A**–**F**). RCM shows an irregular honeycomb pattern of the stratum corneum (**A**), granulosum (**B**–**D**), and spinosum (**E**,**F**) of the epidermis, large cells (koilocytes) (arrows), and hyper-reflective dendritic cells (asterisk).

RCM revealed slight irregularity of the normal honeycomb of the stratum granulosum and/or stratum spinosum of the epidermis associated with the presence of increased dimension cells corresponding to koilocytes which are characteristics of HPV infections (Figure 3). A few hyperreflective dendritic cells could be seen in one case (Figure 3). The papillomatous architecture of the epidermis was visible in the superficial sections of the skin where dark areas surrounded the epidermis projections (Figure 3).

**Figure 4:**
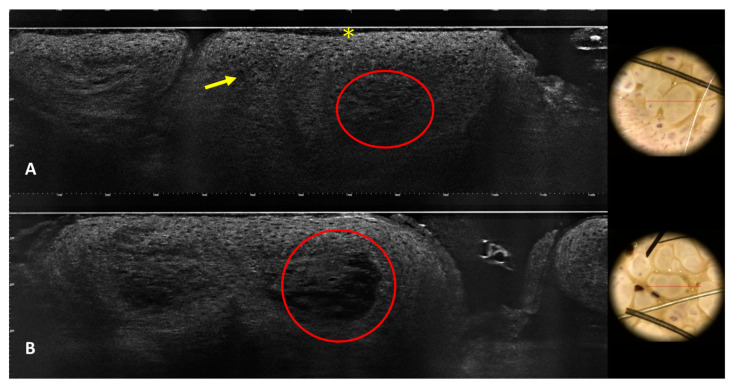
LC-OCT images of condyloma (**A**,**B**). LC-OCT shows vertical and horizontal sections. Hyperkeratosis (asterisk), acanthosis, cells with enlarged nuclei (koilocytes) (arrows), and superficial glomerular vessels (circles) are visible. The red line on dermoscopic images indicates the area imaged by LC-OCT.

**Figure 5:**
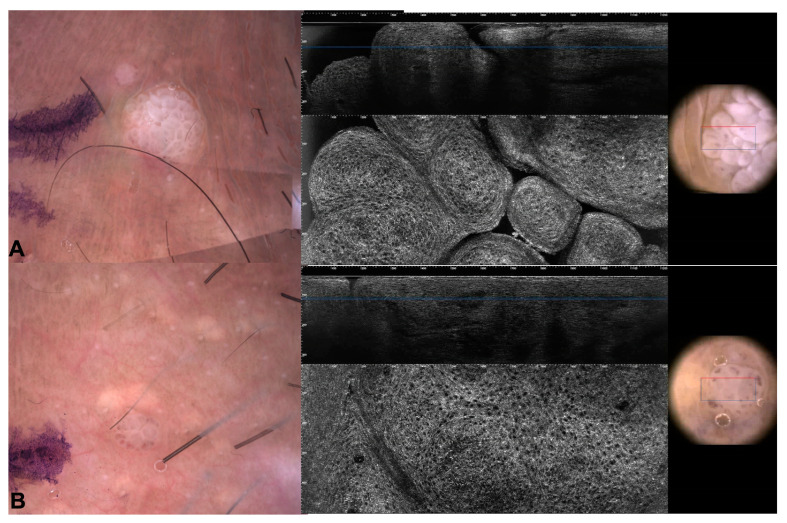
LC-OCT images of two condylomas with respective entire dermoscopic images (**A**,**B**). LC-OCT shows vertical and horizontal sections of condylomas with their corresponding dermoscopic aspects. The rectangle on dermoscopic images indicates the area scanned by LC-OCT. The blue lines indicate the level of horizontal section.

Dilated vessels were visible in the superficial dermis (Figure 2E). In pigmented warts, the basal layer of the epidermis was hyper-reflective due to the pigmentation of basal keratinocytes (Figure 2E). By counting the number of images on vertical stacks, it could also be possible to assess the presence of hyperkeratosis and acanthosis. LC-OCT outlined the architecture of the warts with hyperkeratosis, acanthosis, and enlarged vessels well visible on vertical sections. Keratinocytes were irregular, and few koilocytes seem to be recognizable due to their enlarged nucleus (Figure 4 and Figure 5).

### 3.2. Images of the Imitators of Genital Warts

All clinical imitators of genital warts had dermoscopic examination. Moreover, eleven molluscum contagiosum had LC-OCT examination, three molluscum contagiosum had RCM examination, and lymphangiomas had LC-OCT examination.

**Figure 6:**
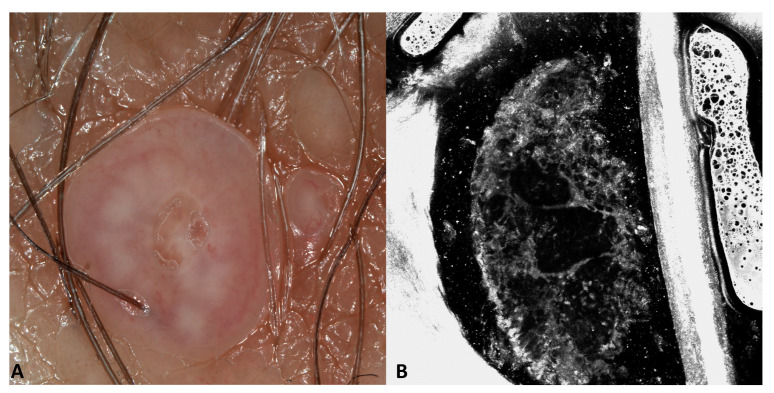
Dermoscopy and RCM images of molluscum contagiosum. Dermoscopy (**A**) shows three pinkish lesions with linear vessels; the largest with a central umbilication and well-visible white globules. RCM (**B**) shows degenerated keratinocytes.

Eleven molluscum contagiosum were in the labia majora, and one was in the pubis (patients number 10, 11, and 12 in Table 1). Dermoscopy revealed pinkish papules in 12 cases, white-yellowish globules confluent in polycyclic areas in 9 out of 12 cases, a central invagination in 8 out of 12 cases, and fine linear vessels mainly at the periphery in all cases. RCM showed roundish, well-circumscribed areas composed of degenerated keratinocytes in all cases (Figure 6).

**Figure 7:**
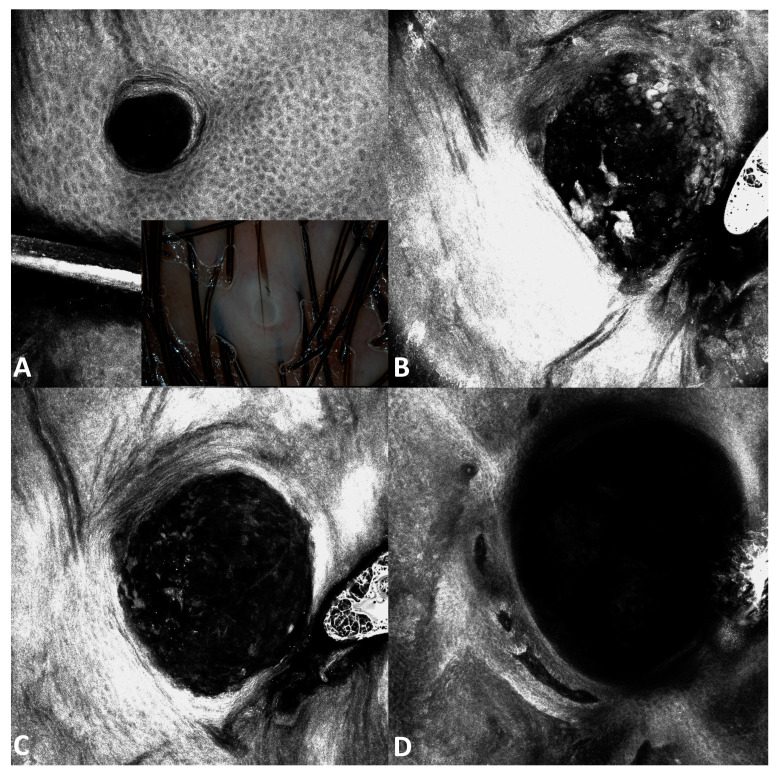
Dermoscopy and RCM images of molluscum contagiosum. Dermoscopy (inset in **A**) shows a pinkish lesion with a central umbilication. RCM shows from the outer to the inner layer a dark hole at the level of the stratum granulosum (**A**), hyperreflective roundish keratinocytes inside a hyporeflective cavity in the spinous layer (**B**), the outer membranes of the degenerated keratinocytes in a deeper layer (**C**), and a large hypo-reflective cavity in the lower part of the epidermis (**D**).

Interestingly, going from the upper part of the affected epidermis to the deeper part: a dark hole was visible at the level of the stratum granulosum, hyperreflective roundish keratinocytes were visible going deeper, and only the outer membrane of the degenerated keratinocytes were appreciated in the inferior part of the epidermis. Going deeper, degenerated cells were not visible anymore and only large hypo-reflective cavities were visible (Figure 7).

**Figure 8:**
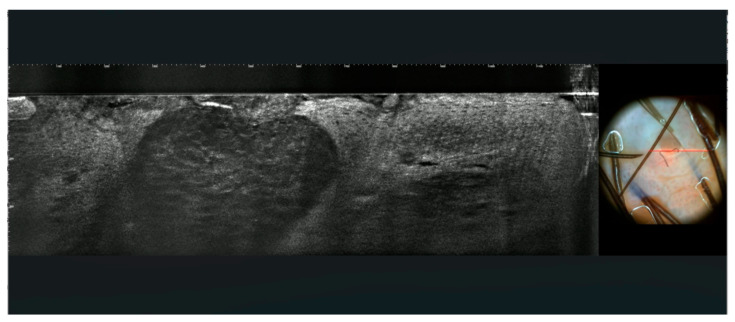
LC-OCT image of molluscum contagiosum. The red line on the dermoscopic image indicates the area imaged by LC-OCT. Large keratinocytes are visible in a well-defined area of the epidermis.

LC-OCT showed hyperplastic epithelium with intraepidermal, well-demarcated craters composed of large hypo-refractive keratinocytes with bright contours in all cases (Figure 8).

**Figure 9:**
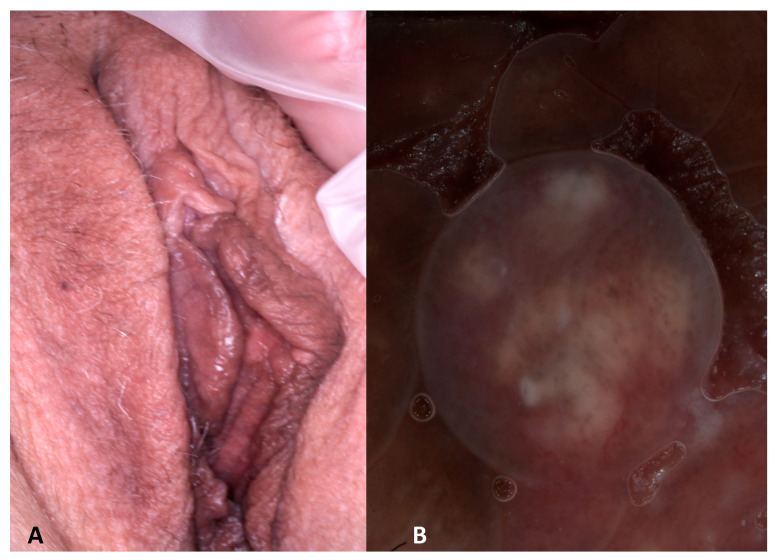
Clinical (**A**) and dermoscopic (**B**) image of a Fordyce’s spot of the labium minor.

The woman with the Fordyce’s spot (patient number 13 in Table 1) showed a flesh-colored papule of the labium minor. Dermoscopy showed whitish-yellowish globules and dotted vessels (Figure 9).

**Figure 10:**
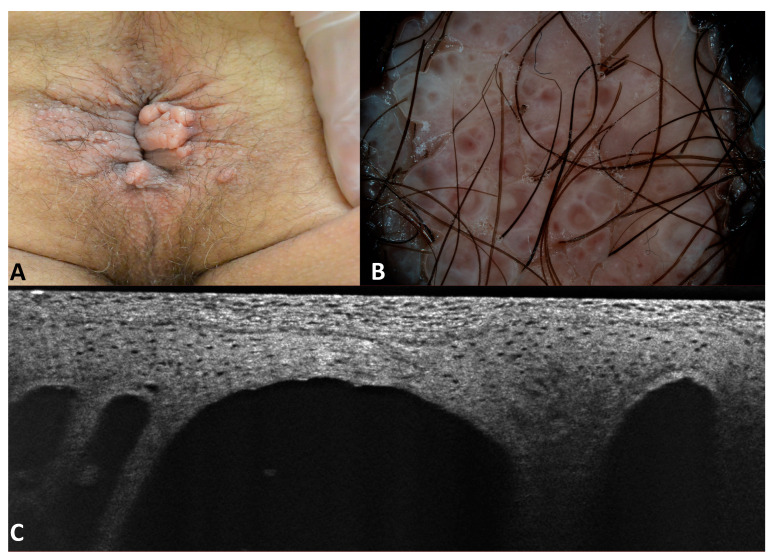
Clinical (**A**), dermoscopic (**B**), and LC-OCT (**C**) image of lymphamgiomas.

The patient with the acquired lymphangiomas (patient number 14 in Table 1) had been suffering from Crohn’s disease for 10 years that had not responded to conventional and biological therapies. He was sent by the gastroenterologist for the evaluation of perianal warts which had been present for a few months. On physical examination, multiple papules confluent into mamellated, translucent plaques were appreciated. Dermoscopy revealed pink lacunae outlined by whitish septa, rare irregular linear vessels, and the characteristic “hypopion sign”. The LC-OCT examination showed well-defined hyporeflective cavities in the superficial dermis with hyper-reflective floating material in the inferior part (Figure 10).

## 4. Discussion

Dermoscopy is a non-invasive diagnostic technique that provides magnified images of the skin at ×10–×30 and has shown value in the examination of pigmented skin lesions. When used to assess non-pigmented skin lesions, it highlights the presence of vessels. The main dermoscopic feature of genital and cutaneous warts is the presence of small structures formed by a central vessel surrounded by a white halo, and depending on the level of protrusion from the skin, it is possible to differentiate a mosaic pattern with relatively flattened and rounded structures, knoblike pattern with shorter and rounded knoblike projections, and fingerlike pattern with separated fingerlike projections similar in size but different in length [4,5]. It is also possible to have a combination of these different patterns in the same lesion or the presence of an unspecific dermatoscopic pattern. Central vessels can be dotted, glomerular, linear, hairpin, curved, annular, and polymorphic [5]. Hemorrhages/extravasation of erythrocytes visible as red/black dots have also been reported, especially in cutaneous warts of the plantar region.

Interestingly, in our study, multiple lesions in the same patient had similar dermoscopic features. As expected, in the majority of our patients, we found central vessels surrounded by a white halo with a mosaic or finger-like (two patients) pattern. One patient had an unspecific pattern with only visible dilated vessels, possibly due to the special location next to the periurethral orifice. Two patients showed pigmented papillomatous warts. Due to their cerebriform pattern, these two lesions were hardly distinguishable from seborrheic keratosis. However, different from seborrheic keratosis, milia-like cysts and comedo-like openings were not visible.

RCM provides horizontal sections of the skin up to the superficial dermis (nearly 250 microns) with a cellular resolution. It is mainly used to detect cutaneous melanoma thanks to its ability to recognize atypical melanocytes as large hyper-reflective roundish or dendritic cells [6]. It has been applied to the diagnosis of some cutaneous infectious and parasitic disorders, allowing a rapid and non-invasive observation of different areas including mucosae in real-time [7]. Although the hand-held probe is more suitable for the examination of sensitive areas like genitalia, some publications showed the feasibility of using the wide probe for imaging genital warts [8].

The diagnosis of condyloma is particularly challenging under RCM because koilocytes, which are the most relevant feature for the diagnosis, can be difficult to recognize. Koilocytes appear in the granular layer or the spinous layer, larger than normal skin keratinocytes with possible large nucleus, and the cytoplasm has a low refractive index [9]. A recent study on 75 patients with histologically proven genital warts found 36% false positive and 21% false negative results based on RCM [9]. Misdiagnosis and missed diagnosis included bowenoid papulosis and squamous cell carcinoma in situ, which can have similar histologic features.

There is a case of topical treatment of genital warts monitored by RCM and conventional OCT [8]. OCT showed papillomatous tumors with a broadening of the epidermis and sometimes keratotic changes including signal-intense dots intratumorally in OCT and subepidermal dark band compatible with inflammation/edema after treatment. RCM showed bright epidermal cells and hypervascularization before treatment and a normal honeycomb pattern after treatment. However, cellular details are not visible on published RCM images.

In our series, RCM showed subtle changes in the epidermis, with a slightly irregular honeycomb pattern that could have been hard to differentiate from normal skin without the clinical context.

LC-OCT is a novel, non-invasive, optical technique commercialized in Europe in 2020, allowing a real-time, vertical and horizontal ‘navigation’ within a skin volume of 1200 µm × 500 µm × 500 µm from the stratum corneum to the dermis at cellular resolution [10]. This device, which integrates a dermoscopic camera allowing precise positioning over the examined areas, has an axial and lateral resolution of 1.1 and 1.3 µm, respectively, that allows the recognition of single cells. It has been mainly used to detect skin carcinomas, particularly basal cell carcinoma [11]. Although conventional OCT does not seem to be a valuable tool for the diagnosis of warts because it can only appreciate their architecture and give no cellular data, LC-OCT has a cell resolution and a potential for the identification of cells with a virus-induced cytopathic effect. In our case series, LC-OCT was able to recognize in vivo the microscopic alterations typical of warts in our series such as hyperkeratosis, mild papillomatosis, and acanthosis. Some koilocytes seem to be recognizable. Moreover, enlarged superficial vessels were well visible. Inside the same lesions, some areas could be better or less visible depending on the level of hyperkeratosis.

Although the identification of mucous membranes and cutaneous warts is generally clinical, non-invasive imaging can help to confirm the diagnosis, saving time-consuming and invasive skin biopsies for histopathological examination. In most cases, dermoscopy shows dilated vessels surrounded by a whitish halo. In clinically and dermoscopically atypical cases, such as pigmented warts, RCM and the new device LC-OCT could identify near histologic features such as the presence of hyperkeratosis, acanthosis, papillomatosis, and enlarged vessels to help the diagnosis. However, the identification of koilocytes, which are the hallmark for the diagnosis of warts, is still difficult with both these techniques.

Genital warts imitators were molluscum contagiosum, lymphangioma, and Fordyce’s spot. Dermoscopy, RCM, and LC-OCT allowed us to easily differentiate these lesions from genital warts.

Molluscum contagiosum is a skin and mucous membrane lesion induced by the moluscipoxvirus [12]. It appears as a dome-shaped, smooth-surfaced, pearly, skin-colored, or white papule that can mimic condyloma in the genital area, especially when it has a hyperkeratotic surface [13,14]. Lesions can be multiple or single, as in our patients. Their central umbilication and their dermoscopic examination may facilitate its diagnosis by revealing white-yellow globules, a possible central keratotic crater, and peripheral vessels in a crown pattern [12]. Interestingly, vessels do not usually cross the center of the structures and this aspect is defined as “red corona” [14]. RCM showed roundish, well-circumscribed areas composed of degenerated keratinocytes that correlate with the characteristic molluscum bodies seen on histopathological examination [7,15]. LC-OCT showed areas of enlarged keratinocytes corresponding to the keratinocytes damaged by the molluscipoxvirus and containing intracytoplasmic viral inclusions (Henderson–Paterson bodies) as described in the literature [16].

The Fordyce’s spot showed whitish-yellowish globules similar to molluscum contagiosum. However, a central umbilication was absent. Fordyce’s spot represents ectopic sebaceous glands and clinically appears as yellowish papules. Dermoscopy shows milky-white-yellowish globules surrounded by wreath-like, non-arborizing vessels. In our case vessels were dotted and did not show the typical “garlands-like” with linear vessels that surround the whitish globules without crossing them [14]. Histologically, the globules correlate with the presence of groups of sebaceous lobules [14].

Lymphangiomas are rare lymphatic malformations. Clinically, they appear as multiple, clustered or scattered, translucent or hemorrhagic vesicules resembling frog eggs composed of dilated lymphatic channels lined by flat endothelial cells located in the superficial dermis of the skin or the mucosa. The color of the vesicles varies according to the sero-hemorrhagic content of the lymphatic channels from tan-yellowish to red-black [17]. The acquired form of cutaneous lymphangiomas is most often found in the axillary, inguinal, and genital areas and it is mainly due to an alteration of the lymphatic circulation with coexisting lymphedema. As in our case, the surface can be warty due to acanthosis and can mimic lesions related to papillomavirus.

In our patient, perianal lymphangiomas were secondary to chronic inflammation of the rectum due to Crohn’s disease. In the vulvar region, lymphangiomas can be consequent to hysterectomy for cervical cancer. The differentiation from genital warts is of paramount importance because we can avoid ineffective and unnecessary treatments, as in our patient.

At dermoscopy, lymphangiomas are characterized by lacunae, generally seen as light brown to yellow-colored roundish areas indicating a fluid content surrounded by pale septa [18]. The hypopyon sign is a dermoscopic clue for the diagnosis and corresponds to a more intense coloration of a part of the lacunae due to dense material that settles at the bottom, corresponding to red blood cells. The intense coloration is often at the inferior area of the lacunae with a horizontal fluid level that can be observed when the patient is standing.

RCM findings have been reported in isolated cases [17] as dark cavities with varying sizes separated by thin septa, which were located in the superficial dermis. The deeper parts of the lacunae were filled with hyper-refractile globular structures of different sizes (20–80 μm). Differently from capillaries and angiomas, there were no circulating blood cells within the lacunas. The non-invasive imaging techniques allowed us to understand that elementary lesions were vesicles and not papules, probably corresponding to lymphangiomas, a diagnosis that was confirmed by histopathology.

Although pearly papules of the genitals and angiokeratomas have also been reported as possible mimickers of genital warts, they were not included in our series because they can be clinically differentiated from genital warts by dermatologists. Pearly papules of the penis or the vagina are anatomical variants that present in histopathology as finger-like protrusions of loose connective tissue covered by normal epithelium [19,20]. They are small flesh-colored papules of the genitalia. Their peculiar symmetric distribution and their confinement to the vestibule or the inner aspects of labia minora in women and the glans contour in men allow their differentiation from genital warts. Moreover, they are softer than condyloma acuminata and dermoscopy shows that the bases of individual papules remain separate, unlike in warts where filiform projections tend to fuse at the base [20].

Angiokeratomas are red-violaceous papules that show, at histopathology, dilated thin-walled blood vessels, lined by a layer of endothelial cells, in the papillary dermis, with acanthosis and hyperkeratosis of the overlying epidermis [21]. At dermoscopy, they show red lacunae and in case of internal thrombosis or trauma lacunae can be black, and hemorrhagic crusts can be present. A multicolored rainbow pattern has also been described at dermoscopy [21]. Their RCM and LC-OCT findings have been recently described by our group: vascular lacunae are visible by both techniques as hyporeflective areas separated by hyperreflective septa, containing multiple small hyperreflective and medium-reflective roundish corpuscles floating inside corresponding to erythrocytes [22]. Angiokeratomas can be solitary or multiple; when they are numerous and extend outside the genital areas, covering the buttock and the lateral aspects of the thigh, Fabry disease, or other lysosomal diseases should be eliminated [22].

Bowenoid papulosis should also be considered in the differential diagnosis of genital warts [23]. It is characterized by pigmented or skin-colored papules and plaques of the genital area. Although it is induced by the HPV virus as genital warts, it is often associated with HPV types 16 and 18, which are the oncogenic high-risk types. It is considered a precancerous condition with a possible rare evolution towards invasive squamous cell carcinoma. Histopathologically, it is characterized by koilocytosis, enlarged polymorphic and hyperchromatic nuclei, and abnormal mitosis and hyper- or parakeratosis with a collection of melanin of various amounts. Although the histopathologic findings are similar to squamous cell carcinoma in situ of the Bowen subtype, cellular atypia is more focal and less pronounced, and follicular involvement is not seen [23]. The most common dermoscopic findings include homogeneous brown pattern, brown and gray dots with linear distribution, and glomerular and dotted vessels [23,24]. RCM has been performed in a few cases [25] and showed acanthosis, irregular honeycomb pattern, and hyper-reflective epidermal dendritic cells probably corresponding to Langerahns cells and melanophages. The described RCM findings do not seem enough specific for a correct differential diagnosis with genital warts and SCC in situ.

RCM and LC-OCT are second-level examinations that can be performed after dermoscopy both in clear-cut cases to increase the level of confidence of dermoscopic diagnosis and in difficult cases to help the clinical diagnosis. These technologies provided additional insights, particularly in identifying specific cellular structures that are not readily apparent with dermoscopy alone. In our series, RCM and LC-OCT were particularly useful in the case of pigmented warts that were hard to identify based only on dermoscopy and could be correctly diagnosed as genital warts under RCM and LC-OCT that showed acanthosis, superficial dilated vessels, and koilocytes. Moreover, RCM and LC-OCT helped to exclude genital warts in the case of their imitators. However, it should be considered that these two new devices are expensive, and the interpretation of their images requires training.

The main limitation of our study is the limited number of lesions included due to the difficulty of acquiring genital images both for the patient’s modesty and the doctor’s difficulty in exploring the genital region. Moreover, we included only lesions that had histological examination, and this procedure is not always performed when the clinical and non-invasive imaging diagnosis is clear.

## 5. Conclusions

In conclusion, this study confirmed the usefulness of dermoscopy in recognizing a precise pattern in warts and showed the potential use of RCM and LC-OCT to add additional findings to the clinical and dermoscopic examination, offering valuable data for future research on larger samples. Imaging examination of lesions that clinically mimic genital warts was divergent from genital warts, helping a non-invasive correct differential diagnosis.

## Figures and Tables

**Table 1:** Clinical features of the lesions.

Patient Number	N. of Lesions	Sex	Age	RCM Images	LC-OCT Images	Site	Clinical Diagnosis	Dermoscopy Diagnosis	Histological Diagnosis
1	2	M	44	yes	yes	inguinal region	SK vs. wart	Wart	wart
2	4	M	50	yes	yes	pubis and penis	SK vs. wart	Wart	wart
3	1	F	40	yes	no	Pubis	SK	SK	wart
4	2	M	50	no	yes	Pubis	wart	wart	wart
5	1	F	35	no	yes	Lip	wart	wart	wart
6	2	M	30	no	yes	pubis	SK	SK	wart
7	3	F	50	no	no	vagina	SK vs. wart	wart	wart
8	3	M	60	no	no	perianal region	wart	wart	wart
9	1	M	61	no	no	urethra	fibroma	Fibroma vs. wart	wart
10	8	F	23	yes	no	labia majora	MC vs. wart	MC vs. wart	MC
11	3	F	43	yes	yes	labia majora	MC vs. wart	MC vs. wart	MC
12	1	M	52	no	no	pubis	MC vs. wart	MC vs. wart	MC
13	1	F	48	no	no	labia minora	wart	MC vs. Fordyce’s spot	Fordyce’s spot
14	multiple	M	51	yes	yes	perianal area	wart	lymphangiectasia	lymphangiectasia

Legend: SK, seborrheic keratosis; MC, molluscum contagiosum.

## Data Availability

The data presented in this study are available on request from the corresponding author.
